# Engineering *Saccharomyces cerevisiae* for Surface Display of a Functional H5 Influenza Virus-Specific Nanobody

**DOI:** 10.3390/microorganisms14061305

**Published:** 2026-06-10

**Authors:** Siqi Xu, Qianmei Xie, Xueer Xie, Xiaomeng Wei, Yangjun Liu, Jiaqi Zhu, Yan Li, Chenying Luo, Ming Liao, Saixiang Feng

**Affiliations:** 1 College of Veterinary Medicine, South China Agricultural University, Guangzhou 510642, China19971656009@163.com (Y.L.);; 2 Meijiang Animal Disease Prevention and Control Center, Meizhou 514000, China; 3 College of Life Sciences, South China Agricultural University, Guangzhou 510642, China; l827528057@163.com; 4 Institute of Animal Health, Guangdong Academy of Agricultural Sciences, Guangzhou 510640, China; 5 College of Animal Science and Technology, Zhongkai University of Agricultural and Engineering, Guangzhou 510225, China

**Keywords:** yeast surface display, *Saccharomyces cerevisiae*, nanobody, fusion orientation, dual-plasmid system

## Abstract

Nanobodies are characterized by their small size, high specificity, and strong affinity, making them promising antiviral agents. In this study, a dual-plasmid yeast surface display (YSD) system based on the *Saccharomyces cerevisiae* a-agglutinin (Aga1p-Aga2p) platform was evaluated for the functional presentation of H5-specific nanobody. To investigate the influence of fusion design on display performance, enhanced green fluorescent protein (EGFP) was fused to Aga2p in two different orientations. Both configurations enabled successful surface display, while the EGFP-AGA2 orientation showed significantly higher display efficiency than AGA2-EGFP (*p* < 0.001). This optimized configuration was subsequently used to display Nb10, a broadly neutralizing nanobody targeting the hemagglutinin (HA) protein of H5 influenza viruses. Indirect ELISA, immunofluorescence, and confocal microscopy confirmed successful surface localization of Nb10, while flow cytometry revealed 22.10% positive cells compared with 0.30% in the negative control (*p* < 0.001). In hemagglutination inhibition (HI) assays, the YSD-Nb10 strain exhibited an HI titer of 3log2, whereas no detectable HI activity was observed in the control strain. Collectively, these results demonstrate the feasibility of displaying a functional H5-specific nanobody using a dual-plasmid YSD system and highlight the importance of fusion orientation for efficient surface presentation, providing preliminary practical guidance for optimization of YSD applications.

## 1. Introduction

Influenza A viruses (IAVs) pose a persistent threat to both the poultry industry and global public health [1,2]. Continuous genetic evolution and antigenic drift of H5 influenza viruses have resulted in the emergence of genetically and antigenically distinct variants, posing significant challenges to vaccine development and disease control [1,3]. Hemagglutinin (HA), the major surface glycoprotein of influenza viruses, plays a central role in viral attachment and entry and is the primary target of neutralizing antibodies. However, frequent antigenic variation within HA, particularly around the receptor-binding region, facilitates viral immune escape and reduces the effectiveness of conventional antibody-based interventions [4,5]. Although several conserved regions remain relatively stable, these epitopes are often structurally constrained and poorly accessible to conventional antibodies. Consequently, alternative antiviral agents with improved epitope accessibility are needed.

Nanobodies (VHHs), derived from camelid heavy-chain-only antibodies, have emerged as promising antiviral agents due to their small size, high stability, and strong binding affinity [3,6,7]. Compared with conventional antibodies, nanobodies can access cryptic or recessed epitopes that are often inaccessible to larger antibody formats and can be readily expressed in microbial systems. These properties make nanobodies particularly attractive tools for biotechnology, diagnostics, and antiviral applications [6,8,9,10,11,12,13]. As previously reported, Nb10 is a broadly neutralizing nanobody against H5 virus that targets a conserved region of HA and exhibits promising antiviral activity [14]. Nb10 was selected as the model nanobody for this study because its binding and neutralizing activities against H5 influenza viruses have been well characterized, making it a suitable benchmark for assessing the performance of the yeast surface display system. Nevertheless, their functional characterization has predominantly relied on soluble expression and purification, which can be labor-intensive and may limit throughput, scalability, and real-time functional screening.

Yeast surface display (YSD) has emerged as a versatile microbial biotechnology platform for protein engineering, antibody screening, and functional characterization. Among available systems, the *Saccharomyces cerevisiae* Aga1p–Aga2p platform is one of the most widely used due to its compatibility with eukaryotic protein folding and flow cytometry-based high-throughput analysis [15,16,17,18,19]. In this system, Aga1p is covalently anchored to the yeast cell wall, while Aga2p is linked to Aga1p through disulfide bonds and serves as the carrier for heterologous proteins displayed on the cell surface [18,19]. Fusion of a target protein to Aga2p enables surface localization in a properly folded and accessible form, thereby facilitating downstream binding and functional analyses. Despite these advantages, conventional YSD systems commonly rely on genomic integration of *AGA1*, which may restrict expression flexibility and limit optimization of display efficiency.

Therefore, we evaluated a dual-plasmid YSD system in *Saccharomyces cerevisiae*, in which Aga1p and Aga2p were independently expressed from separate plasmids for recombinant protein display. Using enhanced green fluorescent protein (EGFP) as a reporter, different fusion orientations were compared, revealing that display efficiency was strongly influenced by fusion design and identifying an optimized configuration for surface presentation. This configuration was subsequently applied to display Nb10, a broadly neutralizing anti-H5 nanobody. Surface localization and biological activity of the displayed Nb10 were confirmed by indirect ELISA, immunofluorescence, confocal microscopy, flow cytometry, and hemagglutination inhibition (HI) assays. These results demonstrate the feasibility of functional nanobody display using a dual-plasmid YSD system and reveal the critical role of fusion orientation in display efficiency. The findings provide a basis for optimizing yeast surface display and support future applications in nanobody engineering and influenza biotechnology.

## 2. Materials and Methods

### 2.1. Gene Retrieval and Plasmid Construction

The DNA sequences of *Saccharomyces cerevisiae* S288C genes *TRP1* (NC_001136.10), *LEU2* (NC_001135.5), *AGA1* (NC_001146.8), and *AGA2* (NC_001139.9) were retrieved from the GenBank database. DNA sequences and plasmid constructs were designed using SnapGene software (version 8.0). The pYSD1 vector contained the *AGA1* and *TRP1* genes, whereas the pYSD2 vector contained the *AGA2* and *LEU2* genes. The plasmids pYSD1-AGA1, pYSD2-AGA2-EGFP, and pYSD2-EGFP-AGA2, together with the Nb10-AGA2 gene fragment, were obtained from General Biosystems (Chuzhou, Anhui, China). In the fusion constructs, EGFP and Nb10 were linked to AGA2p or the V5 tag through a flexible (G_4_S)_3_ linker. The recombinant plasmid pYSD2-Nb10 was constructed by homologous recombination. All primers were listed in Table 1. Detailed information on all plasmids used in this study were provided in Table 2.

### 2.2. Yeast Transformation and Construction of Dual-Plasmid Display Strains

*Saccharomyces cerevisiae* INVSc1 cells (Table 3) were streaked from −80 °C stocks onto YPD agar plates and incubated at 30 °C for 2–4 days. A single colony was inoculated into 3 mL YPD medium and cultured overnight at 30 °C with shaking, followed by expansion in 30–50 mL YPD until OD_600_ reached 0.6–0.8. Cells were harvested by centrifugation (3000 rpm, 5 min), washed with sterile deionized water, and resuspended in 1× LiAc solution to prepare competent cells.

Yeast transformation was performed using the LiAc/PEG method. Briefly, competent cells were mixed with pYSD1-AGA1, carrier DNA, PEG solution, and LiAc solution, followed by incubation at 30 °C for 30 min and heat shock at 42 °C for 30 min. Cells were then collected, resuspended in sterile water, and plated onto SD/-Trp (synthetic defined medium lacking tryptophan) agar plates containing 30 µg/mL kanamycin. After incubation at 30 °C for 2–4 days, positive transformants harboring pYSD1-AGA1 were obtained by PCR.

Subsequently, pYSD2-AGA2-EGFP (C-terminal fusion), pYSD2-EGFP-AGA2 (N-terminal fusion), and pYSD2-Nb10-AGA2 were individually transformed into the recombinant INVSc1 pYSD1-AGA1 strain using the same method. Transformants were selected on SD/-Trp/-Leu (synthetic defined medium lacking tryptophan and leucine) agar plates supplemented with 30 µg/mL kanamycin and 100 µg/mL ampicillin and incubated at 30 °C for 2–4 days. This procedure yielded the dual-plasmid display strains INVSc1 pYSD1-AGA1/pYSD2-AGA2-EGFP and INVSc1 pYSD1-AGA1/pYSD2-EGFP-AGA2, as well as the Nb10-expressing yeast strain INVSc1 pYSD1-AGA1/pYSD2-Nb10-AGA2. For clarity, recombinant yeast strains were abbreviated as follows: INVSc1 pYSD1-AGA1 as YSD-AGA1; INVSc1 pYSD1-AGA1/pYSD2-AGA2-EGFP as YSD-AGA2-EGFP; INVSc1 pYSD1-AGA1/pYSD2-EGFP-AGA2 as YSD-EGFP-AGA2; and INVSc1 pYSD1-AGA1/pYSD2-Nb10-AGA2 as YSD-Nb10. The same abbreviations are used hereafter. The positive transformants were verified by PCR and sequencing. Detailed information on bacterial and yeast strains used in this study were provided in Table 3.

### 2.3. Indirect Elisa for Detection of Surface Display in Yeast Strains

To evaluate the performance of the constructed YSD system, recombinant yeast strains YSD-AGA2-EGFP, YSD-EGFP-AGA2, and YSD-Nb10 were included as experimental groups, while YSD-AGA1 served as the negative control. Single colonies were inoculated into 15 mL SD/-Trp/-Leu liquid medium containing 30 µg/mL kanamycin and 100 µg/mL ampicillin (experimental groups) or SD/-Trp medium containing 30 µg/mL kanamycin (control group), and cultured at 30 °C with shaking (250 rpm) for 24 h. The cultures were adjusted to an OD_600_ of 0.5, harvested by centrifugation, washed, and resuspended in induction medium containing 2% galactose as the sole carbon source. Specifically, recombinant strains harboring both plasmids were cultured in selective synthetic complete medium lacking tryptophan and leucine (SC/-Trp/-Leu), whereas the control strain was maintained in SC medium lacking tryptophan only (SC/-Trp). No glucose was included during the induction phase. Induction was carried out at 28 °C with shaking (250 rpm) for 48–72 h. After induction, cells were collected, washed three times with PBS, and resuspended to an OD_600_ of 2.0 for subsequent analysis.

The prepared cell suspensions were then subjected to ELISA-based analysis. High-binding 96-well ELISA plates (Yeasen, Shanghai, China) were used for coating. In each column, 25 µL of yeast–PBS suspension was added to the first well along with 75 µL of coating buffer, while wells 2–8 received 75 µL coating buffer only. Serial twofold dilutions were performed by transferring 25 µL from the first well to each subsequent well with gentle mixing until the eighth well. Finally, 25 µL was discarded from the eighth well. Plates were sealed and incubated at 4 °C overnight or at room temperature for 2 h. After coating, wells were blocked with 300 µL of 3% BSA solution for 1 h at room temperature. The plates were then incubated with mouse anti-V5 tag monoclonal antibody diluted 1:3000 in PBS (100 µL per well) for 1 h. Following incubation, wells were washed three times with 300 µL of 0.5% PBST (5 min per wash). Subsequently, HRP-conjugated goat anti-mouse IgG diluted 1:5000 in PBS (100 µL per well) was added and incubated for 1 h at room temperature. After three additional washes with 0.5% PBST, 100 µL of TMB substrate solution was added to each well and incubated in the dark for 30 min. The reaction was terminated by adding 100 µL of stop solution. Absorbance was measured at 450 nm using a microplate reader. The obtained data were recorded and analyzed to qualitatively assess the presence and relative level of surface-displayed proteins in the yeast display system.

### 2.4. Fluorescence Microscopy and Flow Cytometry Analysis of Yeast Surface Display

For EGFP detection, 10 µL of the resuspended yeast suspension was mounted on a glass slide, covered with a coverslip, and examined under a fluorescence microscope. For Nb10 display analysis, 100 µL of yeast suspension was incubated with mouse anti-V5 tag monoclonal antibody at 4 °C overnight or at room temperature for 2 h. Cells were then washed three times with PBS and incubated with Alexa Fluor 488-conjugated goat anti-mouse IgG at room temperature in the dark for 1 h. After washing three additional times, cells were resuspended in PBS, and 10 µL of the suspension was applied to a glass slide for fluorescence microscopy observation. Fluorescence signals were used to evaluate the surface expression of the fusion protein. Recombinant yeast strains YSD-AGA2-EGFP, YSD-EGFP-AGA2, and YSD-Nb10 were included as experimental groups, while YSD-AGA1 served as the negative control.

For confocal microscopy, yeast cells of YSD-Nb10 after indirect immunofluorescence staining were resuspended in PBS, and 15 µL of the suspension was mounted on a glass slide and covered with a coverslip without introducing air bubbles. Samples were then examined using a confocal laser scanning microscope to visualize the distribution of fluorescence on the yeast cell surface.

For flow cytometry analysis, yeast cells of YSD-Nb10 after indirect immunofluorescence staining were collected, washed, and resuspended in PBS prior to analysis. A total of 1 × 10^6^ cells were acquired per sample using a flow cytometer. Instrument parameters were set as follows: forward scatter (FSC) = 50, side scatter (SSC) = 50, and FITC channel = 8. Data were recorded and analyzed to quantify the proportion of fluorescent cells and evaluate surface display efficiency.

### 2.5. Hemagglutination Inhibition (Hi) Assay of Yeast-Displayed Nb10

The hemagglutination inhibition (HI) assay was performed using a 96-well V-bottom microtiter plate. Wells 1–10 were used as test wells, well 11 served as the red blood cell (RBC) control, and well 12 served as the antigen control. The YSD-AGA1 served as the negative control. Briefly, 25 µL of PBS was added to each well. Then, 25 µL of yeast–PBS suspension (OD_600_ = 2.0) was added to the first well and mixed thoroughly. Twofold serial dilutions were performed across wells 1–10 by transferring 25 µL of the mixture sequentially, with the final 25 µL discarded from well 10. Subsequently, 25 µL of 4 hemagglutinating units (HAU) from inactivated antigen H5-rD8 was added to wells 1–10 and well 12, while 25 µL of PBS was added to well 11. The plate was gently mixed and incubated at room temperature for 30 min to allow antigen–nanobody interaction. After incubation, 25 µL of 1% chicken RBC suspension was added to each well. The plate was gently agitated for 1 min and then incubated at room temperature for 40 min. The results were visually assessed when the RBC control well (well 11) formed a distinct button at the bottom of the well. The HI titer was defined as the highest dilution of the yeast suspension that completely inhibited hemagglutination.

## 3. Results

### 3.1. Construction of a Dual-Plasmid Yeast Surface Display System

**Figure 1:**
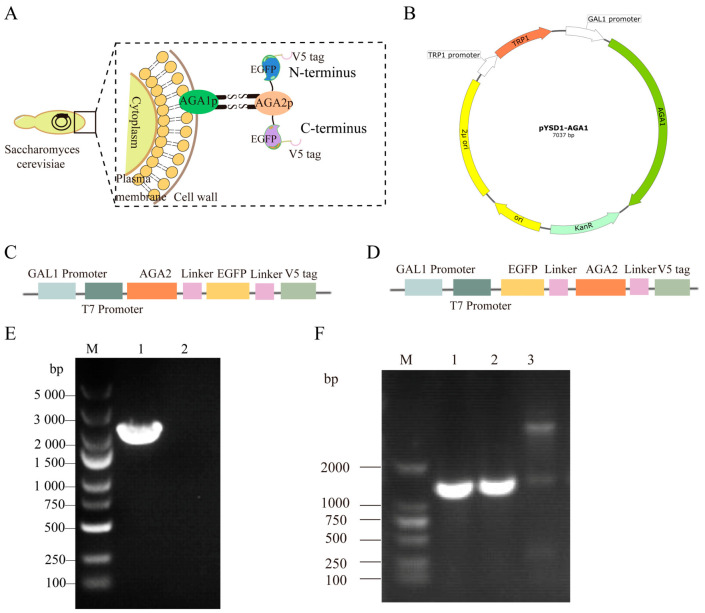
Construction of a dual-plasmid yeast surface display system. (**A**) Schematic representation of the yeast surface display (YSD) system. AGA1p is anchored to the yeast cell wall and covalently linked to AGA2p through disulfide bonds. EGFP is fused to AGA2p either at the C-terminus or the N-terminus, enabling surface display of the fluorescent protein on yeast cells. (**B**) Schematic map of pYSD1-AGA1. This plasmid harboring the *TRP* selection marker and the *AGA1* gene. The size of the plasmid is indicated in the diagrams. (**C**,**D**) Schematic illustrations of the AGA2–EGFP and EGFP–AGA2 fusion constructs. Orange and yellow blocks represent the gene of *AGA2* and *EGFP*, respectively. Promoters and tags are indicated by green blocks of different shades, while linkers are shown as pink blocks. (**E**,**F**) Agarose gel electrophoresis analysis of PCR products confirming the presence of *AGA1* gene (**E**) and the fusion genes *AGA2–EGFP* and *EGFP–AGA2* (**F**). The observed band sizes are consistent with the expected target fragments. M, DNA marker. In (**E**), lane 1 shows the expected size of the *AGA1* fragment (~2300 bp), and lane 2 represents the negative control (untransformed INVSc1 yeast strain). In (**F**), lanes 1 and 2 correspond to the expected sizes of AGA2–EGFP and EGFP–AGA2 (both ~1300 bp), respectively, while lane 3 represents the negative control (INVSc1 yeast strain transformed with pYSD-AGA1 only).

To investigate the feasibility of a dual-plasmid YSD configuration, Aga1p and Aga2p were expressed from separate plasmids in *Saccharomyces cerevisiae*. As illustrated in Figure 1A, AGA1p was anchored to the yeast cell wall and formed disulfide bonds with AGA2p, enabling the presentation of fusion proteins on the cell surface. Based on this system, two fusion configurations were constructed, in which EGFP was linked to either the C-terminus or the N-terminus of AGA2p. Figure 1B shows the plasmid map of pYSD1-AGA1, whereas Figure 1C,D show schematic representations of the pYSD2-AGA2-EGFP and pYSD2-EGFP-AGA2 expression cassettes, respectively. The pYSD1-AGA1 plasmid carries the *AGA1* gene and the *TRP1* selectable marker, whereas the pYSD2 constructs encode EGFP fused to either the N- or C-terminus of Aga2p. The recombinant plasmids were sequentially transformed into the *Saccharomyces cerevisiae* INVSc1 strain. Briefly, competent INVSc1 cells were first transformed with pYSD1-AGA1, followed by transformation with either pYSD2-AGA2-EGFP or pYSD2-EGFP-AGA2. PCR amplification was performed to verify the successful construction and transformation of the target genes. As shown in Figure 1E, PCR amplification using the AGA1-F/R primer pair yielded a DNA fragment of approximately 2300 bp corresponding to AGA1, whereas no band was observed in the negative control. In Figure 1F, amplification with the AGA2-EGFP-fused-F/R primers produced fragments of approximately 1300 bp corresponding to AGA2–EGFP and EGFP–AGA2. The sizes of all plasmids were consistent with the expected designs. No specific amplification was detected in the negative control. These results confirm the correct construction of the plasmids and successful introduction of the target genes into the yeast system. The primer sequences are listed in Table 1.

### 3.2. Surface Display of Aga2-Egfp and Egfp-Aga2 Fusion Proteins in Yeast Strains

**Figure 2:**
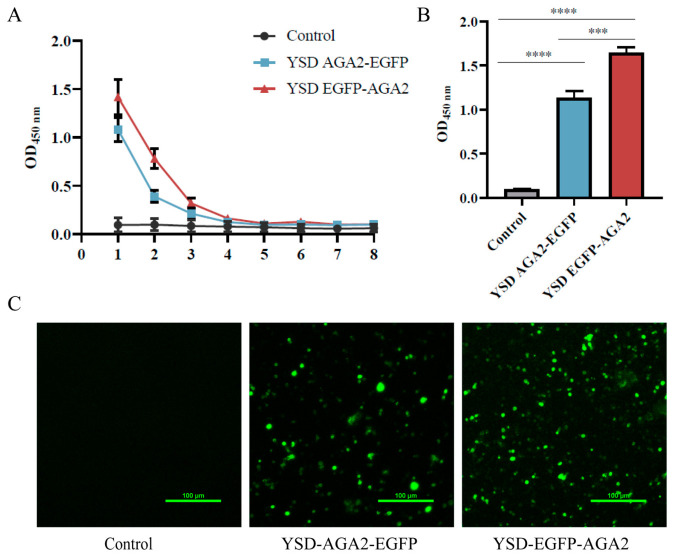
Indirect ELISA and fluorescence analysis of yeast surface-displayed EGFP. (**A**) Indirect ELISA analysis of yeast cells displaying AGA2–EGFP or EGFP–AGA2. Serial dilutions (wells 1–4) showed a gradual decrease in OD_450_ values in both experimental groups, while no obvious signal was detected in the negative control. (**B**) Comparative analysis of indirect ELISA results. The OD_450_ values of both experimental groups showed significantly higher than the control (*p* < 0.0001), with YSD-EGFP-AGA2 exhibiting a stronger signal than YSD-AGA2-EGFP (*p* < 0.001). (**C**) Fluorescence microscopy analysis of EGFP expression on the yeast cell surface. Both experimental groups display clear fluorescence signals compared with the negative control. A slightly higher proportion of fluorescent cells was observed in the YSD-EGFP-AGA2 strain than in the YSD-AGA2-EGFP strain. Statistical significance is indicated as follows: *** *p* < 0.001, **** *p* < 0.0001.

Surface expression of the AGA2-EGFP fusion proteins was evaluated by indirect ELISA and fluorescence analysis (Figure 2). As shown in Figure 2A, both YSD-AGA2-EGFP and YSD-EGFP-AGA2 groups exhibited a gradual decrease in OD_450_ values across serial dilutions (wells 1–4), indicating a typical titration-dependent binding pattern, whereas no obvious signal was detected in the negative control group. Compared with the negative control, both EGFP-displaying yeast strains showed highly significant increases in ELISA signals, indicating that EGFP-fused proteins were successfully displayed on the cell surface. These results confirm the reliability of the assay and demonstrate successful detection of the V5-tagged EGFP on the yeast cell surface. Notably, both experimental groups showed significantly higher OD_450_ values compared with the negative control (*p* < 0.0001, Figure 2B). And, the YSD-EGFP-AGA2 strain exhibited markedly higher signal intensity than the YSD-AGA2-EGFP strain (*p* < 0.001, Figure 2B). Fluorescence analysis further supported these findings (Figure 2C). Both experimental groups displayed clear fluorescence signals compared with the negative control, confirming successful expression of EGFP. Although the overall fluorescence intensity between the two groups appeared comparable, a slightly higher number of fluorescent cells was observed in the YSD-EGFP-AGA2 strain than in the YSD-AGA2-EGFP strain based on microscopic examination, which is in agreement with the ELISA results. Together, these data demonstrate that both fusion orientations enable successful surface display, with the EGFP-AGA2 configuration showing improved display performance.

### 3.3. Construction of the Nb10-Based Yeast Surface Display Plasmid

**Figure 3:**
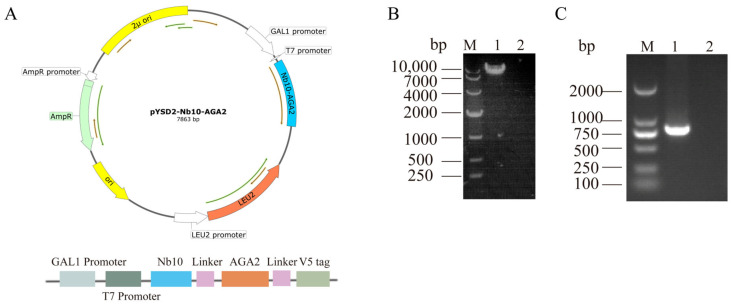
Construction of the recombinant plasmid pYSD2-Nb10-AGA2. (**A**) Plasmid map of the recombinant vector pYSD2-Nb10-AGA2 and schematic representation of the Nb10-AGA2 expression cassette. The Nb10 gene fragment was fused to the N-terminus of Aga2p and cloned into the pYSD2 vector containing the LEU2 selectable marker. In the expression cassette shown below the plasmid map, the Nb10 and AGA2 coding sequences are depicted as blue and orange blocks, respectively, separated by a linker sequence. The corresponding genes or functional elements are labeled within each block. (**B**) PCR analysis of the linearized pYSD2 vector. A DNA fragment of approximately 7300 bp was observed. (**C**) PCR analysis of the Nb10-AGA2 fragment. A product of approximately 800 bp was obtained. The observed band sizes were consistent with the expected fragments.

Based on the combined results of indirect ELISA and fluorescence microscopy, the pYSD1-AGA1/pYSD2-EGFP-AGA2 system was selected as the optimal YSD platform, in which the target protein is fused to the N-terminus of AGA2p. To enable surface display of Nb10 using this configuration, the Nb10 coding sequence was fused to *AGA2* and inserted into the pYSD2 vector by seamless cloning, generating the recombinant plasmid pYSD2-Nb10-AGA2, which carries the LEU2 selectable marker (Figure 3A). In the schematic representation of the expression cassette shown in Figure 3A, Nb10 and AGA2 are indicated by blue and orange blocks, respectively. For plasmid construction, the pYSD2 backbone was first amplified using primers YSD2-AGA2-F/R (Table 1) to obtain a linearized vector of approximately 7300 bp (Figure 3B). In parallel, the Nb10-AGA2 fragment was amplified using Nb10-AGA2-F/R primers (Table 1), yielding a product of approximately 800 bp (Figure 3C). The observed fragment sizes were consistent with the expected design, thereby confirming the successful preparation of both the vector backbone and the insert for subsequent cloning. Following ligation and transformation, the recombinant plasmid pYSD2-Nb10-AGA2 was successfully constructed, as verified by PCR and sequencing. This construct subsequently served as the foundation for further expression and functional analysis of Nb10 in the YSD system.

### 3.4. Surface Display of Nb10 on Yeast Cells

**Figure 4:**
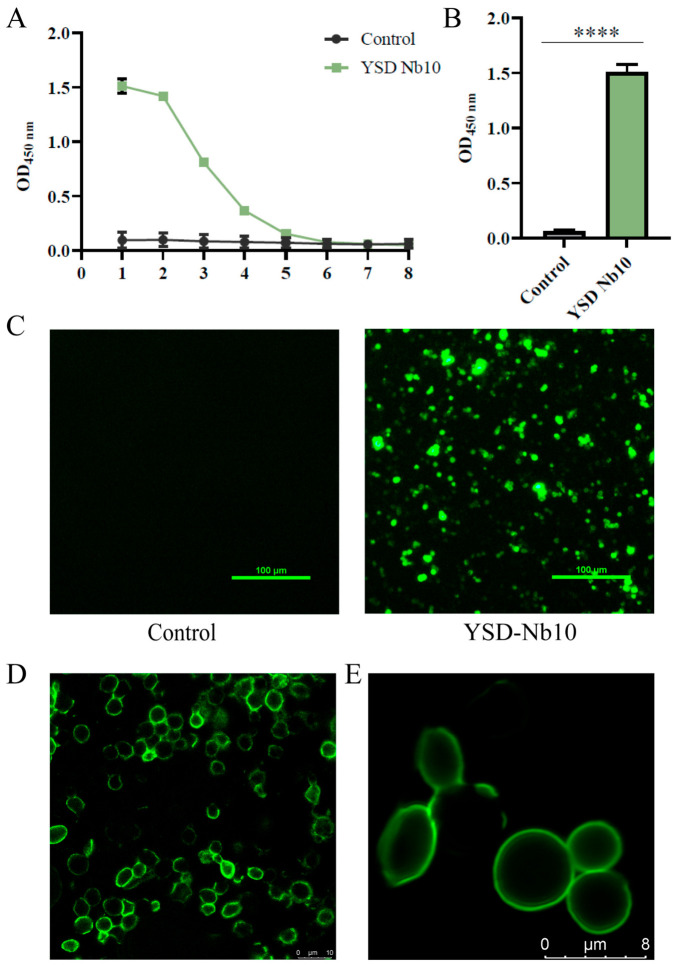
Detection and visualization of surface-displayed Nb10 by indirect ELISA and fluorescence microscopy. (**A**) Indirect ELISA analysis of the YSD-Nb10 yeast strain. The OD_450_ values decrease progressively across serial dilutions (wells 1–5), while the negative control shows background-level signals. (**B**) Comparative analysis of indirect ELISA results. The YSD-Nb10 group showed a significantly higher OD_450_ value than the negative control (*p* < 0.0001), supporting the surface expression of V5-tagged Nb10. (**C**) Immunofluorescence analysis of Nb10 surface display. The YSD-Nb10 strain displays prominent fluorescence, whereas negligible signal is observed in the control group. (**D**,**E**) Confocal microscopy analysis of YSD-Nb10 yeast cells. Fluorescence was predominantly localized at the cell periphery, supporting successful surface display of Nb10. Images were acquired at 200 × (**D**) and 600 × (**E**) magnification. Statistical significance is indicated by **** (*p* < 0.0001).

To verify successful surface display of the V5-tagged Nb10–AGA2 fusion protein, indirect ELISA was performed using the YSD-Nb10 yeast strain (Figure 4). The OD_450_ values of serially diluted samples (wells 1–5) showed a gradual decrease in all experimental groups, whereas no obvious signal was detected in the negative control (Figure 4A), indicating the reliability of the assay. Based on the OD_450_ values of the first well, a highly significant increase in signal was observed in the YSD-Nb10 group compared with the negative control (*p* < 0.0001, Figure 4B), demonstrating that the V5-tagged Nb10–AGA2 fusion protein was successfully displayed on the yeast cell surface. Indirect immunofluorescence staining further confirmed these findings. As shown in Figure 4C, strong fluorescence signals were observed in the YSD-Nb10 group, whereas no detectable signal was observed in the negative control.

To further examine the localization of the displayed protein, confocal laser scanning microscopy was performed. As shown in Figure 4D,E (200× and 600× magnification, respectively), fluorescence signals were predominantly distributed along the periphery of the yeast cells, consistent with cell surface localization. These observations confirm that the Nb10–AGA2 fusion protein was successfully displayed on the yeast cell wall in the YSD-Nb10 strain. Together, these results demonstrate the successful surface display of Nb10 in the yeast surface display system.

### 3.5. Quantitative Analysis of Nb10 Surface Display and Hemagglutination Inhibition Activity

**Figure 5:**
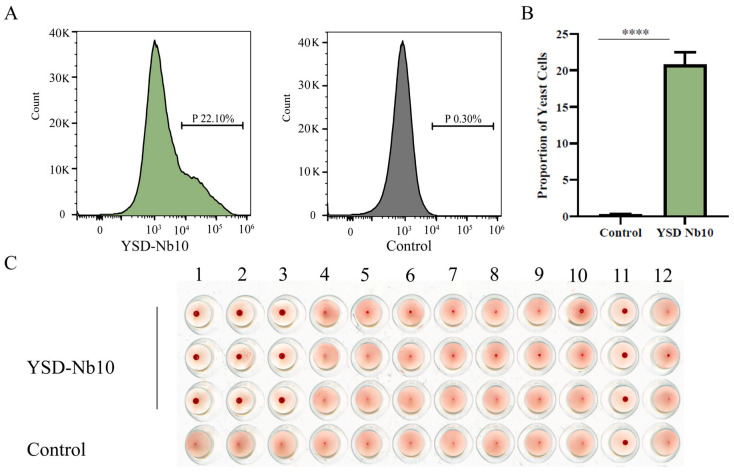
Flow cytometry analysis of surface-displayed Nb10 and hemagglutination inhibition (HI) assay. (**A**) Percentage of fluorescent yeast cells determined by flow cytometry after immunolabeling. The YSD-Nb10 group showed a substantially higher proportion of fluorescent cells (22.10%) compared with the control strain (YSD-AGA1, 0.3%), indicating successful surface expression of V5-tagged Nb10. (**B**) Statistical comparison of fluorescent cell percentages. The YSD-Nb10 group exhibited a significantly higher proportion of positive cells than the control (*p* < 0.0001). (**C**) HI assay of Nb10. The HI assay was performed using inactivated H5 (rD8) influenza virus. The control group showed no detectable HI activity, whereas the YSD-Nb10 strain exhibited clear inhibition with a titer of 3log2. **** *p* < 0.0001 indicates an extremely significant difference.

To quantitatively assess Nb10 surface display, flow cytometry was performed to determine the proportion and fluorescence intensity of labeled yeast cells (Figure 5A,B). The YSD-Nb10 strain exhibited a substantially higher percentage of fluorescent cells compared with the control strain (YSD-AGA1) (*p* < 0.0001). Approximately 22.10% of cells in the YSD-Nb10 group were positively stained, whereas only 0.3% positivity was observed in the control group, indicating efficient surface expression of Nb10. To further evaluate the biological activity of the displayed Nb10, a hemagglutination inhibition (HI) assay was conducted using the inactivated vaccine of H5-rD8 (Figure 5C). The control strain showed no detectable HI activity, while the YSD-Nb10 strain exhibited clear inhibition of hemagglutination, with an HI titer of 3log2. In this assay, wells 11 and 12 were designated as the negative and positive controls, respectively. These results demonstrate that Nb10 is not only successfully displayed on the yeast cell surface but also retains functional activity against the H5 influenza virus.

## 4. Discussion

In this study, we evaluated a dual-plasmid yeast surface display (YSD) platform in *Saccharomyces cerevisiae* and demonstrated its applicability for functional presentation of an H5 influenza virus-specific nanobody. By systematically comparing different fusion orientations, we further showed that display efficiency is strongly influenced by the spatial arrangement of the fusion protein on the yeast surface. The optimized configuration enabled successful surface localization of Nb10 and preserved its biological activity, as demonstrated by hemagglutination inhibition (HI) assays. Nb10 was selected as a proof-of-concept model because its antigen-binding and antiviral activities had been previously established [14]. The use of a well-characterized nanobody enabled assessment of the display system while reducing uncertainties associated with the characterization of a newly identified nanobody. Collectively, these findings demonstrate the feasibility of functional nanobody display using a dual-plasmid YSD system and provide preliminary guidance for optimization of yeast-based display systems, although validation using additional proteins will be necessary to establish their broader applicability.

Traditional Aga1p–Aga2p systems generally rely on genomic integration of *AGA1*, while only the Aga2p fusion construct is plasmid-borne [20,21,22]. Although such systems are widely used, they may provide limited flexibility for coordinated optimization of scaffold and target protein expression. This study evaluated a dual-plasmid yeast surface display (YSD) configuration in which Aga1p and Aga2p fusion proteins were expressed from separate plasmids in *Saccharomyces cerevisiae*. Rather than introducing a fundamentally new display platform, this work focused on assessing the applicability of such a configuration for nanobody presentation and examining factors that influence display performance. The dual-plasmid architecture provides a modular framework that may facilitate future optimization through independent manipulation of Aga1p and Aga2p expression levels, promoter engineering, or plasmid design. However, further studies will be required to determine whether these potential advantages translate into improved display efficiency or broader applicability compared with existing YSD systems.

A key finding of this study is that fusion orientation significantly affects display efficiency. Both AGA2-EGFP and EGFP-AGA2 constructs successfully mediated surface display; however, the EGFP-AGA2 configuration exhibited higher display efficiency. Previous studies have suggested that the spatial positioning of displayed proteins relative to the yeast cell wall can strongly influence folding, steric accessibility, and functional presentation [23,24,25]. Fusion of the target protein to the N-terminus of Aga2p may reduce steric hindrance near the cell surface and improve epitope accessibility. In the present system, the EGFP-AGA2 configuration may therefore better support proper folding and surface exposure of the fusion protein. Importantly, the same configuration also enabled successful display of the H5-specific nanobody Nb10, suggesting that careful consideration of fusion orientation may be beneficial for optimizing Aga2p-mediated nanobody display. These findings highlight the importance of rational fusion design when developing yeast-based display applications. Beyond the proof-of-concept demonstration presented here, yeast surface display systems have previously been applied in areas such as affinity maturation, screening, biosensing, and molecular recognition [16,22,26,27,28,29,30,31,32]. Although these applications were not investigated in the present study, the successful display of a functional H5-specific nanobody suggests that similar directions may be explored in future work. Further experimental validation will be required to determine the suitability of the current configuration for such applications.

It is noteworthy that the HI activity observed for yeast-displayed Nb10 was lower than that previously reported for the purified soluble nanobody. In our earlier study, soluble Nb10 exhibited a HI-IC_50_ of 0.10 ± 0.00 µg/mL and typically achieved HI titers of approximately 12–13 log2 against H5 influenza viruses [14]. In contrast, the YSD-Nb10 strain displayed a HI titer of 3 log2 in the present study. However, these values are not directly comparable because the level of Nb10 displayed on the yeast surface could not be accurately determined. Moreover, the measured HI activity of the yeast-displayed format is influenced not only by the intrinsic antiviral potency of Nb10 but also by factors such as display efficiency, surface accessibility, steric constraints, and the proportion of cells expressing detectable nanobody on the surface. Therefore, the primary significance of the HI assay in this study is to demonstrate that Nb10 retained biological activity following surface display, rather than to provide a quantitative assessment of its antiviral potency relative to the soluble nanobody. Future studies incorporating quantitative determination of surface-displayed nanobody levels and appropriate benchmark controls will be necessary to enable more rigorous comparisons between soluble and yeast-displayed formats.

Nevertheless, several limitations should be acknowledged. Flow cytometry analysis showed that approximately 22.10% of yeast cells displayed Nb10 on the surface, which appears lower than that reported in some previous YSD systems [33]. Such heterogeneity is commonly observed in yeast display platforms and may result from differences in plasmid copy number, transcriptional activity, secretion efficiency, or protein folding. Further optimization of promoter strength, secretion signals, codon usage, or cultivation conditions may improve display uniformity and expression efficiency. In addition, because the present platform relies on the coordinated expression of Aga1p and Aga2p fusion proteins from two separate plasmids, plasmid instability or loss during cell growth and galactose induction may also contribute to the observed heterogeneity. Cells that lose either plasmid would be expected to exhibit impaired surface display despite remaining viable within the population. Although plasmid retention was not directly assessed in this study, future work should investigate plasmid stability throughout the induction process and evaluate alternative strategies, such as genomic integration or optimized plasmid maintenance systems, to improve display consistency. Furthermore, although indirect ELISA, immunofluorescence analysis, and flow cytometry provided consistent evidence of successful surface display, the molecular integrity of the Aga2p fusion protein was not directly evaluated. Biochemical characterization, such as Western blot analysis of cell wall-associated protein fractions, would be valuable for confirming the expected molecular weight of the displayed fusion protein and excluding potential proteolytic cleavage during expression and surface anchoring. Moreover, although indirect ELISA, immunofluorescence analysis, hemagglutination inhibition assays, and flow cytometry collectively demonstrated antigen recognition and biological activity of the displayed nanobody, the present study did not include quantitative measurements of binding affinity or binding kinetics. Future studies should therefore incorporate surface plasmon resonance (SPR), flow cytometry-based binding titration, or other quantitative approaches to more comprehensively characterize the interaction between the displayed nanobody and its target antigen. Furthermore, virus neutralization assays and structural studies will be necessary to evaluate antiviral efficacy and elucidate the molecular basis of antigen recognition. Finally, the platform was validated using a single anti-H5 nanobody as a proof-of-concept model. Consequently, it remains unclear whether the orientation-dependent effects observed in this study are specific to Nb10 or are broadly applicable to other nanobodies and heterologous proteins. Additional studies incorporating diverse protein formats will be required to determine the generalizability of these findings and to further evaluate the robustness of the display configuration.

In conclusion, we developed and evaluated a dual-plasmid YSD configuration in *Saccharomyces cerevisiae* and demonstrated that fusion orientation critically influences surface display efficiency. The optimized system enabled functional display of the broadly neutralizing nanobody Nb10 while preserving antigen-binding activity against H5 influenza virus. These findings provide useful guidance for rational engineering of yeast display systems and evaluate a versatile microbial platform for nanobody-based biotechnology applications.

## 5. Statistical Analysis

Data were analyzed using GraphPad Prism 8.0. Results are presented as mean ± standard error (SE). Statistical significance between groups was determined using Student’s *t*-test. All experiments were performed in triplicate with consistent results. Significance levels were defined as follows: *p* < 0.05 (*), *p* < 0.01 (**), *p* < 0.001 (***), and *p* < 0.0001 (****); ns indicates no significant difference.

## Figures and Tables

**Table 1:** Primers used in this study.

Primer	Sequences (5′-3′)
AGA1-F	GAACCCTAAAGGGAGCCCCCGATTTAG
AGA1-R	GTCTCCCCGCGCGTTAGTGAATAG
AGA2-EGFP-fused-F	CTAGCAGCTGGAATATTAAGC
AGA2-EGFP-fused-R	TGTGCAATTGCGTAAACTCG
Nb10-AGA2-F	GCTTCAGTTTTAGCACAGGTTCAGTTACAAG
Nb10-AGA2-R	TCAGCGGGTTTAAACTCACGTAGAATCGAG
YSD2-AGA2-F	GTTTAAACCCGCTGATCTGATAACAAC
YSD2-AGA2-R	TGCTAAAACTGAAGCAATAACAG

**Table 2:** Plasmid used in this study.

Names	Relevant Characteristic(s)	Source
pYSD1-AGA1	Yeast surface display vector expressing Aga1p as the cell wall anchor protein	General Biosystems
pYSD2-AGA2-EGFP	Yeast expression vector encoding a C-terminal EGFP fusion to Aga2p for display efficiency evaluation	General Biosystems
pYSD2-EGFP-AGA2	Yeast expression vector encoding a N-terminal EGFP fusion to Aga2p for display efficiency evaluation	General Biosystems
pYSD2-Nb10	Yeast expression vector encoding Nb10–Aga2p fusion for nanobody surface display	This research

**Table 3:** Bacterial and yeast strains used in this study.

Names	Relevant Characteristic(s)	Source
*E. coli* DH5α	*deoR endA1 gyrA96 hsdR17 (rk-mk+) recA1 relA1 supE44 thi-1* Δ(lacZYA-argF) U169 Φ80lacZ ΔM15F-λ -	Vazyme
INVSc1	MATa *his3*Δ*LEU2 TRP1-289 URA3-52*/MATα *his3*Δ*LEU2 TRP1-289 URA3-52*	This research
INVSc1 pYSD1-AGA1 (YSD-AGA1)	*Saccharomyces cerevisiae* INVSc1 strain carrying pYSD1-AGA1 expression vector for surface display	This research
INVSc1 pYSD1-AGA1/ pYSD2-AGA2-EGFP (YSD-AGA2-EGFP)	*Saccharomyces cerevisiae* INVSc1 strain carrying pYSD1-AGA1 and pYSD2-AGA2-EGFP expression vector for surface display	This research
INVSc1 pYSD1-AGA1/ pYSD2-EGFP-AGA2 (YSD-EGFP-AGA2)	*Saccharomyces cerevisiae* INVSc1 strain carrying pYSD1-AGA1 and pYSD2-EGFP-AGA2 expression vector for surface display	This research
INVSc1 pYSD1-AGA1/ pYSD2-Nb10-AGA2 (YSD-Nb10)	*Saccharomyces cerevisiae* INVSc1 strain carrying pYSD1-AGA1 and pYSD2-Nb10-AGA2 expression vector for Nb10 surface display	This research

## Data Availability

The original contributions presented in this study are included in the article. Further inquiries can be directed to the corresponding authors.
